# How productive emergency physicians work: a qualitative interview study of clinical workflow in the emergency department

**DOI:** 10.1186/s13049-026-01638-w

**Published:** 2026-06-12

**Authors:** Moa Berglund, Leila Piroozam, Joëlle Golmann, Katarina Bohm, Malgorzata Kopaczek-Styczen, Li Jin Yang, Anders Sondén, Jenny Liu, Kristian Ängeby

**Affiliations:** 1 Department of Emergency Medicine, Stockholm South General Hospital (Södersjukhuset), Stockholm, Sweden; 2 Department of Clinical Science and Education, Södersjukhuset, Karolinska Institute, Stockholm, Sweden; 3 Department of Health Sciences, The Swedish Red Cross University, Huddinge, Sweden; 4 Department of Surgery, Stockholm South General Hospital (Södersjukhuset), Stockholm, Sweden

**Keywords:** Emergency department, Physicians, Efficiency, Productivity, Workflow, Time management, Qualitative research, Interview

## Abstract

**Background:**

Operational efficiency in the emergency department (ED) is crucial for minimizing patient wait times, preventing overcrowding, and optimizing resource utilization. Although systemic inefficiencies in ED operations have been extensively studied, the understanding of individual physician-level work practices that support high productivity is limited. This study aimed to explore how productive emergency physicians structure their work in order to provide insights into physician-level practices that may support clinical workflow and overall departmental efficiency.

**Methods:**

This study employed a thematic analysis informed by Braun and Clarke’s reflexive approach to analyze semi-structured interviews with eight high-throughput emergency physicians at Stockholm South General Hospital (Södersjukhuset) in Stockholm, one of Sweden’s largest emergency hospitals, between March and May 2021. Five predefined topic areas (prioritization, communication, examination, laboratory and imaging modalities and medical record management), guided data collection and the initial organization of the dataset, after which themes were developed inductively through an iterative and collaborative analytic process.

**Results:**

Thematic analysis developed six themes characterizing how productive emergency physicians structured their workflow: initial situational awareness, parallel progression, cognitive offloading, anticipatory communication, selective resource utilization and pragmatic responsiveness.

**Conclusion:**

This study provides insights into work practices that may enhance productivity in emergency medicine, with the potential to be reinforced through targeted training. Integrating these approaches into physician education could help reduce patient wait times, prevent overcrowding, and optimize resource utilization, thereby improving overall workflow. Further research is needed to explore their broader implications for clinical decision-making, healthcare efficiency, and patient safety.

**Supplementary Information:**

The online version contains supplementary material available at 10.1186/s13049-026-01638-w.

## Background

A common topic of discussion in the emergency department (ED) is how to increase efficiency [1–3], defined here as the combination of individual and systemic outcomes. Improving efficiency is essential not only for reducing lengthy wait times but also for preventing overcrowding and mitigating the risk of medical errors [4].

Prolonged wait times in the ED present multiple challenges. Particularly, overcrowding has been linked to increased patient mortality and negative impacts on flow and resource utilization [5, 6]. Additionally, excessive wait times are among the most frequently cited patient complaints [7], contributing to frustration and reduced trust in the healthcare system.

While much attention is directed toward extrinsic, department-level factors contributing to inefficiencies, there is limited guidance on how emergency physicians can improve their own work processes [8]. Prior work, such as the analysis by Azan et al. of the “How I Work Smarter” series, has highlighted productivity strategies among emergency physicians, but these largely reflect self-reported experiences and include both professional and personal domains [9]. Other studies investigating productivity have primarily relied on Relative Value Units (RVUs) per hour, a billing-based metric widely used in the United States but less applicable in European settings [2]. In this study, we therefore define productivity more generally as individual physician output per unit of time, a definition that is both suitable for the Scandinavian context and more readily comparable across healthcare systems [10].

A focus on individual physician strategies for higher productivity in the ED is important for several reasons. Observations of different physicians reveal substantial variability in output per time unit within the ED, yet much of this variability remains unexplained [3, 4, 9–11]. If productivity can be cultivated, identifying the specific attributes and strategies employed by high-performing physicians is highly valuable. This raised our research question: How do the most productive physicians structure their work? To address this question, we explored the work practices of high-throughput physicians in our ED.

## Methods

### Study design

The study was informed by a constructivist paradigm, recognizing reality as being socially and contextually constructed [12]. To explore how productive emergency physicians structured their work, we collected qualitative data through semi-structured interviews and used thematic analysis informed by Braun and Clarke’s reflective approach [13]. The study was performed in accordance with good clinical practice and research as per the Helsinki Declaration [14]. All the participants provided verbal informed consent prior to the interviews.

### Setting and participants

In this study, we employed purposive sampling to recruit the most productive physicians working in the Emergency Department at Stockholm South General Hospital (Södersjukhuset), Stockholm, Sweden, between March and May 2021. Södersjukhuset has one of the busiest emergency departments in Sweden. In 2021, the total number of visits was 82,000, with an admission rate of 35%. During the study period, the median length of stay in the emergency department was 5 h and 46 min. During this time, the medical staff comprised 32 specialist physicians and 47 resident physicians.

Physician productivity was operationalized as the average number of patients seen per hour, adjusted for non-clinically active time such as lunch breaks. To identify these physicians, we analyzed productivity data for doctors working in the Emergency Department. The QlikView^®^ tool (Qlik Technologies Inc., King of Prussia, PA, United States) was used to assess the number of patients seen by each physician. The shifts included in the study were as follows: 21:00–08:30, 07:30–15:30, 10:00–18:00, 13:00–21:30, and 16:00–23:30. Only patients initially examined by the physician were included, (i.e. hand-overs were excluded) and a 30-minute break was excluded from the total time. The patient-to-hour ratio was then calculated, and the twelve physicians with the highest ratios were contacted. Of these, the first eight who agreed to participate were included in the study.

The concept of information power guided the sample size [15]. Given the narrow research question, clearly defined participant group, and richness of the interview data, eight interviews were considered sufficient to provide depth and nuance in exploring the work practices employed by high-throughput emergency physicians.

### Data collection

The interviews were conducted either in person or via video conferencing via Microsoft Teams (Microsoft Corporation, Redmond, WA, USA) or Zoom (Zoom Video Communications Inc., San Jose, CA, USA). All the interviews were facilitated by a single interviewer (LP), who at the time was employed as an emergency medicine resident at Södersjukhuset in Stockholm.

The interviews followed a semi-structured format, guided by open-ended questions (Appendix A), with additional follow-up questions tailored to participants’ responses, ensuring relevance to the topic under discussion. The Participants were not provided with the interview questions in advance. The interview guide was organized around five topic areas based on the research team’s clinical experience and existing literature on ED workflow [8–10]: prioritization, communication, examination, laboratory and imaging modalities, and medical record management. These topic areas were used to ensure broad coverage of key aspects of emergency department workflow. Each interview lasted approximately one hour. All interviews were conducted in Swedish, audio-recorded, and transcribed verbatim by the interviewer. Quotations included in the manuscript were translated into English by MB and reviewed by KÄ and JG to ensure that the meaning of the original quotations was retained.

### Reflexivity

The research team brought different levels and types of experience in emergency care to the study, ranging from longstanding specialist experience at the study site to more limited clinical experience during medical training. These differing positions shaped both the questions asked and the interpretations developed during analysis. As an example, the interviews were conducted by a researcher (LP) who worked as a resident physician in the same emergency department as the participants. Although this professional familiarity likely created an environment of trust and openness during the interviews, facilitating richer conversations, it may also have led to implicit assumptions or shared understandings not being explicitly articulated, possibly affecting the clarity or depth of certain responses. Reflexive discussions within the team allowed different professional and experiential perspectives to inform how the data was understood and how the themes were developed. To enhance transparency, we describe the analytic steps explicitly regarding how themes were constructed.

### Analysis

We conducted a thematic analysis informed by Braun and Clarke’s reflexive approach [13]. The five predefined topic areas guided data collection and the initial organization of the dataset but were not treated as final themes.

Analysis began with repeated reading of the transcripts to support familiarization with both individual interviews and the dataset as a whole. During this phase, initial analytic observations were made in relation to how productive emergency physicians described structuring their workflow. We then coded the material, initially organizing data extracts in relation to the five topic areas while remaining open to patterns that extended across them.

Through iterative and collaborative analysis, we subsequently developed themes inductively across the dataset. This involved moving back and forth between coded extracts, the full dataset, and developing interpretations in order to review, refine, define, and name themes. Although coding drew on participants’ explicit descriptions of their workflow, the final themes were developed at an interpretive level and were intended to capture broader patterns of meaning rather than simply summarize interview topics. The final themes should therefore be understood as analytic outputs developed through interpretation, rather than as direct reflections of the interview guide structure.

The transcripts were analyzed by two researchers (LP and MB) and interpretations were discussed by four researchers (LP, MB, JG and KÄ). Rather than aiming for coding reliability [13], the focus was on achieving depth and nuance in these discussions. The final themes were refined through an iterative and collaborative analysis process. Only one of the researchers (LP) could link transcripts to individual physicians.

The results are presented with direct quotations from participants, with minor edits for clarity, such as the removal of interviewers’ filler words.

## Results

The study included four emergency physicians and four residents in emergency medicine (six males and two females), aged between 30 and 65 years. Physicians were selected based on their high patient productivity rates. Their professional experience ranged from 5 to 25 years. The individual productivity of the included physicians ranged from 0.9 to 1.1 patients per hour (PPH) with a median of 1.0 PPH. Four additional physicians who had been invited but were not included showed similar productivity.

The thematic analysis developed six key themes characterizing how productive emergency physicians structured their workflow. Each theme is illustrated by representative quotations from the participants.

### Initial situational awareness

This theme captures how physicians created an early overview of patients, team capacity, and overall departmental situations at the beginning of the shift. The physicians described this as an important first step in structuring their workflow, as it helped them identify which patients required more immediate attention, which cases were likely to demand more thorough investigation, and where early action might be needed:

I start by scrolling through the list to see what types of patients I currently have. I identify which patients are likely to require further investigation and which ones I can probably handle more quickly. It usually takes a minute or so to obtain an understanding of what kind of issues patients have. This way, I can also identify the sicker patients. (Physician 1).

They also emphasized the importance of consulting nurses early to help prioritize care and manage workload more effectively:

When I begin my shift, I review the patient list and then consult with the nurse about the patients—asking who they recommend I see first and what actions they suggest. If the patient list is overwhelming, such as having 15 patients, a quick round of assessments is often effective. (Physician 3).

For some, situational awareness at the start of a shift extended beyond patient prioritization to include assessing their team´s level of experience. This was described as a way of setting appropriate expectations, facilitating coordination, and ensuring that all members contributed effectively to patient care. In this sense, situational awareness concerned not only patients, but also the immediate organizational conditions of work:

I ask everyone how much they have worked, meaning their level of experience. It is largely our responsibility as doctors to ensure that the team start is effective. I take a report from the outgoing colleague. I begin by reviewing the entire patient list and go through each patient, checking the documentation, including vital signs, lab results, and any other information I may need. (Physician 5).

## Parallel progression

This theme refers to how the physicians moved several patients forward in parallel by initiating time-dependent tasks early and using waiting time to advance other cases. Rather than managing one patient at the time in a strictly sequential manner, they described organizing workflow so that processes such as laboratory testing and radiology could begin early, while they continued assessing or following up other patients. In this way, waiting time did not remain idle but became part of the workflow, helping to reduce avoidable delays and maintain steady patient flow:

I try to initiate several tasks simultaneously. That’s the only way to make it work. I’m good at prioritizing—deciding what to start now that could have an effect in a few hours. It’s not just about ticking things off one by one, but rather managing multiple tasks. After all, this is an emergency department, not an outpatient clinic. I think that’s a key factor. I do not go beyond what needs to be done. I enjoy having multiple tasks in progress at the same time. It’s crucial when working in the emergency department to be able to handle several things concurrently. (Physician 7).

This approach allowed them to continue managing other patients while waiting for results and ensured a smoother admission and discharge process:

I typically assess multiple patients simultaneously. After a brief discussion with each patient, I initiate the necessary actions, such as ordering tests, which usually take approximately 45 minutes to process. During this time, I can return to the patient to follow up on their condition, as there is no immediate need for a full assessment. If I have a younger patient who can be managed quickly, I will attend to them while awaiting the test results for the other patients. Therefore, I do not work sequentially from one patient to the next; rather, I manage several patients at once. (Physician 8).

## Cognitive offloading

The theme captures the use of structured routines and cognitive aids to reduce mental workload and to support consistency in patient care, documentation, and decision-making. Rather than relying solely on memory in a setting marked by interruptions, multiple competing tasks, and high information load, physicians described creating supports and repeatable routines that helped them gather information, document findings, and make decisions in a consistent way. Some used structured note-taking methods to streamline documentation:

I print out the most recent medical note with the correct information and then make annotations on it. This becomes my draft note for that patient (Physician 1).

Others relied on pre-saved protocols to facilitate clinical decision-making:

I have protocols saved in my login, and I frequently refer to them. (Physician 4).

These practices appeared to serve several functions at once: they reduced the mental effort required to keep track of multiple details, helped participants avoid repeated questions or duplicated work, and supported a more systematic approach to chart review, history-taking, examination, and documentation. Several physicians described following predefined algorithms or mnemonic structures, not only to ensure thoroughness, but also to stabilize workflow under time pressure:

I follow an algorithm that I go through. I check vital signs, diagnoses, and get an overall impression. Then I review the medication list. If the patient is multimorbid, I check a previous medical note. I also look at lab results and the ECG. After that, I go to the patient. I usually do not make notes during patient visits. I use the SOKRATES mnemonic method when I’m with the patient. I prefer to see the patient only once. I decide right away whether the patient needs to be admitted or can go home. Afterward, I dictate when I’m finished and know what the plan for the patient is. I never save dictations until later. (Physician 2).

Physicians also emphasized the importance of taking regular breaks as part of sustaining cognitive performance throughout the shift. In this sense, cognitive offloading was not only about external aids such as protocols or notes, but also about preserving the capacity to think clearly and work consistently over time:

I always take my break, regardless of the situation in the Emergency Department. Afterward, I return and continue working efficiently. I also take short pauses, stepping away from the workstation to have a cup of coffee. Many colleagues mention that they have not had time for their break, but it is essential to prioritize it. Taking a break does not impact the overall functioning of the Emergency Department, but it is crucial for maintaining personal well-being and sustaining performance throughout the shift. (Physician 6).

## Anticipatory communication

This theme captures how physicians communicated proactively with both patients and colleagues in ways that appeared to reduce misunderstandings, interruptions, and inefficiencies in the ED. Rather than simply exchanging information, they anticipated what needed to be conveyed to both patients and colleagues to prevent misunderstandings, streamline patient flow, and optimize care delivery in the ED.

### Patient communication

The physicians emphasized the importance of providing patients with clear, structured information to minimize uncertainty and unnecessary interruptions:

A less effective communication approach may be attributed to failure in informing patients about what they are waiting for or not clearly outlining the plan of action. This lack of involvement can lead patients to be unaware of what is expected or pending. It is essential to ensure that patients receive adequate information so that they do not ask numerous questions, such as “What happens next?“, or “when will my transport arrive” (Physician 1).

The physicians emphasized the value of structuring patient interactions to ensure that all necessary details were gathered from the start, preventing the need for repeated questioning and enhancing productivity:

I begin by taking the patients history and performing the examination, and I make an effort to follow the chart template even while I am with the patient, ensuring that I gather the necessary information to complete the medical record during the initial visit. This way, I avoid having to return and ask questions such as, ‘How is your smoking habit?’ or ‘What is your occupation?’ or collect details such as weight and height later. If I determine that the patient needs imaging, I directly ask for their weight and height so that I have all the necessary data at hand. My goal is to ensure that only one visit is needed. (Physician 1).

In addition, they suggested that proactively addressing potential patient concerns could improve patient flow by reducing unnecessary follow-ups and minimizing return visits due to misunderstandings about red flags or when to seek further medical attention:

To the patient, I ask open-ended questions, listen, and as soon as I am finished, I provide a summary and inform the patient, who may offer input or not. Once that is done, we are finished. I also ask targeted questions. This process takes me approximately 5–7 minutes. (Physician 6).

When communicating with the patient, I ask open-ended questions. I believe in the classic approach of not interrupting the patient. Everything you need to know; the patient has already told you in the first two minutes. Studies show that it takes just one minute. (Physician 8).

### Team communication

At the team level, the physicians suggested that by maintaining positive team dynamics and ensuring that colleagues feel supported, they could prevent friction and inefficiencies before they impact patient care. They also emphasized the importance of clear and considerate communication in fostering a supportive and inclusive environment, enhancing collaboration, and contributing to a more efficient workflow in the ED.

A positive work environment contributes to a more enjoyable and engaging workplace. While it should never come at the expense of patient care quality, fostering a supportive and inclusive team dynamic is essential. A relaxed and collaborative atmosphere enhances both individual and team performance. I prioritize verbal communication to ensure clarity. If team members are overwhelmed, I assist them in prioritizing urgent and critical tasks. (Physician 3).

At the colleague level, the physicians described how direct and structured communication with nursing staff helped optimize workflows, especially in a resource-limited environment:

Our problem is that we have a shortage of nursing staff. Clear directions help make their work easier, which in turn makes our work easier. (Physician 6).

The physicians also highlighted that being direct yet respectful helped maintain a functional team dynamic, allowing tasks to be completed more efficiently:

I strive for direct communication. If the nursing staff says they do not have time or are overwhelmed with tasks, I ask, ‘Should you or I contact the head nurse?’ I am direct and clear: ‘This needs to be done now. If you cannot do it, we need to ask for help. With the team quick check-ins are very effective. Everyone gains better insight, and it leads to a stronger team. (Physician 8).

## Selective resource utilization

The emergency physicians demonstrated a strategic approach to limiting examinations and resource utilization. They focused on prioritizing what was clinically justified and directly relevant to the patient’s immediate condition, distinguishing between urgent actions and those that could be deferred. Recognizing these limitations is essential for optimizing clinical decision-making and maintaining efficient resource management:

I strive to limit investigations to what is pertinent in the Emergency Department, focusing only on what is necessary and clinically justified. (Physician 2).

The physicians tailored the investigations to the patients’ symptoms, ensuring that only the most relevant tests were ordered and interpreted. They described using targeted investigations – such as laboratory tests, diagnostic imaging, and ECG – specifically to rule out emergent conditions rather than performing broad, unnecessary screenings. Moreover, they applied the same principle of selectivity to documentation, ensuring that medical notes remained concise and focused only on essential information:

I document what is relevant. I try to keep it simple. I write concise referrals, including only what is necessary and relevant. I limit what I document to essential information. Only relevant details are recorded. A good medical note is a maximum of one page. This can serve as a general principle. One should be able to understand, in retrospect, the reasoning behind the decisions made and why they were made. (Physician 2).

In managing complex cases, physicians prioritized acute conditions that required immediate intervention while deferring chronic issues to inpatient care when admission was necessary:

I assess the reason for their visit and address the acute symptoms first. If admission is required and the patient has multiple issues, I prioritize urgent concerns, while chronic conditions can be managed on the ward. I aim to focus on immediate needs and limit the scope of treatment in the emergency setting. Given the high volume of geriatric patients with multiple comorbidities, it is not feasible to address everything at once. Chronic issues can be dealt with on the ward, as we must focus on the acute conditions in the emergency department. (Physician 7).

## Pragmatic responsiveness

This theme captures how physicians adapted their work to rapidly changing circumstances, including shifts in patient condition, new clinical information, or evolving healthcare demands. In addition to adjusting their decision-making, many of the physicians described taking on tasks typically assigned to nurses or support staff when necessary. This flexibility was often driven by staffing shortages and the need to maintain workflow efficiency under high team workloads:

If there are numerous care-related tasks, I sometimes try to assist by handling some of them myself, as I can perform these tasks equally well. For example, if a patient I have attended, requires something to eat or needs to use the toilet, I may take care of that, ensuring that the nursing staff can focus on the tasks where I require assistance (Physician 1)

A consistent pattern also emerged in how these physicians sustained their productivity throughout the shift. Toward the end of their working hours, they often choose to manage less complex cases, minimizing handovers and reducing disruptions during shift changes:

I make an effort to adhere to the patient list. Toward the end of my shift, I may cherry-pick certain cases to help reduce the list, particularly when I am nearing the end of my shift and can ensure that I complete the management of a patient to avoid an incomplete handover. (Physician 2).

Finally, physicians adapted their approach to individual patient needs, adjusting their management strategies on the basis of factors such as cognitive impairment and the reliability of patient self-reports:

There is no one-size-fits-all approach for patients; rather, the management plan is based on the severity of the patient’s condition, their level of cognitive impairment, and how much trust can be placed in the patient’s self-reports. In cases involving elderly patients with dementia who are unable to provide a coherent history, one must rely on laboratory tests, contact family members, and conduct a thorough, systematic examination from head to toe. (Physician 8).

## Discussion

This study aimed to explore how productive emergency physicians structure their workflow. Using a qualitative approach, we developed six key themes—*initial situational awareness*, *parallel progression*,* cognitive offloading*,* anticipatory communication*,* selective resource utilization*, and *pragmatic responsiveness*—that offer insights into how productive emergency physicians navigated the demands of emergency care. With the continued rise in patient volume and ED overcrowding, physicians are increasingly required to organize their work in ways that preserve flow under pressure. Although numerous studies have investigated extrinsic, department-level factors influencing ED efficiency, comparatively less is known about physician-level work practices associated with higher productivity. Our findings therefore complement prior literature on emergency physician productivity by highlighting how productive physicians structure their work. Although the PPH for the physicians interviewed in this study (0.9–1.1) appears lower than in comparable studies, e.g. 1.94 in Oskvarek et al. [16] and 1.8 in Nikolla et al. [17], it is important to note that these previous studies exclude teaching hospital sites and shifts where productivity might be impaired by non-patient work. Our findings provide insights into work practices that may support physician productivity and improve workflow in the emergency department. Across several of our themes, a broader pattern was the prevention of bottlenecks. Productive physicians described working in ways that anticipated and contained delays before these disrupted patient flow.

One such strategy was *situational awareness* at the beginning of each shift. Reviewing the patient list, familiarizing oneself with the team, and gaining an overview of departmental activity appeared to create the conditions for this way of working by helping physicians identify which patients required more immediate attention and which time-dependent processes could be initiated early. Our interpretation is that situational awareness throughout the shift—through continued monitoring of patient status, team workload, and clinical priorities—also supported faster decision-making, smoother workflow [18], and timely recognition of clinical deterioration [4]. These findings are consistent with previous research suggesting that increased situational awareness is important for both operational efficiency and patient safety [4, 10, 19, 20]. In this sense, situational awareness was not only an orienting practice in its own right, but also a prerequisite for subsequent workflow management.

*Parallel progression* may be understood as the practical enactment of this logic. Rather than managing one patient at the time in a strictly sequential manner, physicians initiated time-dependent steps early, while continuing to assess or follow up other patients. By organizing work in this way, waiting time could be incorporated into the broader workflow. This interpretation is consistent with previous literature suggesting that minimizing avoidable delays is important for preserving patient flow in the emergency department [18].

When combined with continuous situational awareness, parallel progression may also help prevent the type of downstream batching observed by Feizi et al., where admitting multiple patients simultaneously without integrating them through the full workflow, including timely dispositions and discharges, led to reduced system efficiency and longer patient length of stay as a cost of increased individual productivity [21].  A similar logic was evident in the theme *anticipatory communication*. The participants consistently emphasized the importance of clear communication—both within the team and with patients—and the role of a positive work environment in maintaining a smooth workflow. Physicians described providing patients with structured information about what was happening, what they were waiting for, and what would happen next, which appeared to reduce uncertainty, repeated questions, and unnecessary follow-up. Within the team, anticipatory communication—such as establishing clear plans and preemptively addressing coordination needs—seemed to reduce miscommunications, friction, and coordination delays. These findings are consistent with previous studies [10, 20]. In our interpretation, anticipatory communication within the team is especially relevant in the emergency department, where physicians, according to previous studies, face an average of 11 interruptions per hour—a figure that increases with patient volume and significantly affects workflow and decision-making capacity [4, 22]. These findings align with prior research showing that effective communication, a cohesive team culture, and anticipatory planning enhance workflow, improve efficiency, and reduce medical errors [17, 22].

An important boundary condition is that these strategies are applicable when physicians can distribute attention across multiple patients. In cases of severe instability, such as resuscitation or shock, workflow may necessarily become concentrated on a single patient, thereby limiting opportunities for parallel progression.

Despite the broad endorsement of evidence-based practice, clinical efficiency remains variable, and over-investigation continues to pose a significant barrier to optimal ED throughput [1]. *Selective resource utilization* may be understood as another way of preserving flow. Physicians described focusing investigations on what was clinically relevant and time-sensitive, while avoiding unnecessarily broad work-ups. In our interpretation, this reflects a practical logic of selectivity in which testing, treatment, and documentation are organized around immediate clinical need. Physicians who adopted structured decision-making strategies, including the use of clinical mnemonics such as the PERC rule or the HEART score, appeared to reduce unnecessary testing and shorten patient wait times. These interpretations align with previous literature suggesting that structured decision aids and routine-based cognitive strategies increase diagnostic accuracy, improve workflow efficiency, and reduce healthcare costs [1]. One possible interpretation is that applying evidence-based tools within a selective investigative framework may support flow and productivity in the emergency department by enabling more targeted diagnostic testing and more appropriate use of resources [1]. Our findings also suggest that timely and confident decision-making was important for maintaining patient flow. While it has been shown that residents become more productive for each year of residency [23] and that specialists are more productive than residents [24], strategies to support faster and more confident decision-making may be relevant at any stage of clinical experience.

Productive physicians in our study consistently demonstrated a structured, systematic approach, incorporating personal routines and evidence-based tools to gather clinical information. Our interpretation is that this strategy supports *cognitive offloading*, which may reduce the cognitive load and improve consistency in clinical decision-making. Frequent reference to validated protocols and local guidelines was described as supporting decision-making, and minimizing unnecessary investigations—factors believed to contribute to both quality of care and efficiency [1]. In this sense, cognitive offloading did not primarily concern moving patients forward in parallel or preventing bottlenecks directly, but rather making focused and selective decision-making more manageable under conditions of interruption and time pressure. Previous studies have suggested a steady workflow [23] and completion preference [25] as other forms of cognitive offloading.

Another common strategy among physicians was the intentional scheduling of breaks to preserve energy and sustain performance throughout the shift. Prior studies suggest that physician productivity typically peaks early and declines over the course of the shift, although responsiveness to high-acuity patients is generally maintained [18, 23]. Notably, physicians with the highest patient volumes sustained their throughput later in shifts by prioritizing lower-complexity cases. Additionally, previous studies have indicated that completing care for one’s own patients—rather than handing off cases at the shift-end—may contribute to improved overall efficiency and enhance continuity for incoming staff [18, 26]. At the same time, these work practices should not be assumed to be cost-free for individual clinicians. Several of the strategies described in our material, such as sustained situational awareness, parallel progression, and anticipatory communication, may involve considerable cognitive and organizational effort. The relationship between productivity-oriented work practices, clinician workload, fatigue, and burnout therefore remains unclear and warrants further study.

Another recurring pattern in our material was *pragmatic responsiveness*, reflecting an individual’s ability to adapt to changing systems, roles, and clinical demands. Physicians who demonstrated greater adaptability appeared better equipped to navigate shifting priorities, manage interruptions, and maintain workflow under pressure.

Adaptive performance, which involves embracing organizational change and proactively acquiring new knowledge, remains relatively underexplored in healthcare research. Most studies have traditionally focused on task and contextual performance, often overlooking this important domain. However, emerging literature suggests that adaptive performance is associated with proactive behavior and creativity [27]. Given its relevance in dynamic clinical settings, we believe further research is warranted in this area.

Although admission and discharge decisions were not strongly developed as a distinct topic in our interview material, some participants described organizing work in ways that supported smoother transitions and reduced incomplete handovers. At the same time, several important workflow challenges were not sufficiently elaborated in the dataset. Procedural tasks may represent a different kind of bottleneck, as they often require continuous physician attention, specific resources, and team coordination, making them less amenable to the same preventive strategies. Similarly, supervision of trainees and the management of uncertainty or adverse outcomes are important dimensions of emergency care that may shape workflow, but they were not developed in enough depth in the present material to support stronger conclusions.

In summary, our findings suggest that physician-level strategies—including initial situational awareness, parallel progression, cognitive offloading, selective resource utilization, anticipatory communication, and adaptability by pragmatic responsiveness—may support workflow within the constraints of the emergency department. These individual approaches offer practical insights for improving productivity in emergency care settings.

## Methodological considerations

Several methodological considerations are necessary. First, all participants were recruited from the same emergency department and shared a common organizational context, which may have reduced the diversity of experiences represented in the material. This may limit the transferability of the findings to other settings. In line with a constructivist approach, we sought transferability rather than statistical generalizability [28]. Transferability can be considered in different ways, including applicability to other settings and resonance with readers’ own professional contexts [28]. To support such judgments, we have aimed to provide a clear description of the study setting, participants, and analytic process.

Second, physicians with lower patient throughput were not included. Although contrasting perspectives might have added further analytic breadth, recruiting physicians on the basis of lower productivity was considered ethically sensitive and may have introduced discomfort or perceived stigmatization among those approached.

Third, the study focused on physicians identified as highly productive based on patient throughput. Although this operationalization captured one important dimension of emergency department work, it did not encompass all aspects of emergency physician performance, such as the quality of care, patient safety, management of severely unstable patients, procedural work, supervision of trainees, or the handling of clinical uncertainty and adverse outcomes. The findings should therefore be interpreted as a partial account of productive workflow patterns rather than as a comprehensive account of effective emergency physician performance.

Fourth, the findings are based on interview accounts of work practices rather than direct observation of clinical workflow. As such, they reflect how participants described and made sense of their work, rather than offering an exhaustive representation of what occurred in practice.

Finally, the findings should be understood as situated analytic interpretations rather than neutral or exhaustive representations of emergency physician workflow. The research team brought different levels of familiarity with emergency care and with the local study setting, and these perspectives informed both data generation and interpretation. We have therefore sought to be transparent about these positions and about the interpretive nature of the analytic process [13, 29].

## Conclusion

This study provides insights into work practices used by productive emergency physicians that may support productivity and workflow in the emergency department. The six themes highlight physician-level strategies that could help reduce patient wait times, prevent overcrowding, and optimize resource utilization, thereby improving overall workflow. While these findings suggest areas that could be developed through targeted training, they should be understood as interpretive and contextually situated rather than evidence of universally effective strategies. Their implications for patient safety, clinical outcomes, clinician workload, and overall emergency department efficiency remain unclear and warrant further study.

## Supplementary Information

Below is the link to the electronic supplementary material.

Supplementary Material 1

## Data Availability

Interview transcripts can be obtained upon request.

## Abbreviations

ED: Emergency Department
PPH: Patients Per Hour
ECG: Electrocardiogram
PERC rule: Pulmonary Embolism Rule–out Criteria
HEART score: History, ECG, Age, Risk factors, and Troponin score
