# Supplementary material for: How productive emergency physicians work: a qualitative interview study of clinical workflow in the emergency department
Source: Scand J Trauma Resusc Emerg Med. 2026 Jun 12;34:105. doi: 10.1186/s13049-026-01638-w (PMC13263933; doi:10.1186/s13049-026-01638-w)
Supplement: Supplementary file 1 — Supplementary Material 1 [file 13049_2026_1638_MOESM1_ESM.docx]

**Appendix A

Semi-structured interview questions**

**General:**

- If you were to describe freely, what factors (if any) do you believe make you an effective emergency physician?
- In what ways (if any) do you think your approach to work differs from that of less effective emergency physicians?
- Describe a typical shift for you.
- How do you manage breaks during your shift, and in what ways (if any) do you think this differs from less effective emergency physicians?
- Where and how do you spend your time when working in the emergency department, and in what ways (if any) do you think this differs from less effective emergency physicians?
- In what ways (if any) do you think your personality differs from that of less effective emergency physicians?

**Prioritisation:**

- In what order do you see patients, and in what ways (if any) do you think this differs from less effective emergency physicians?
- In what order do you perform tasks when managing a patient, and in what ways (if any) do you think this differs from less effective emergency physicians?

**Communication:**

- How do you communicate with nursing staff, and in what ways (if any) do you think this differs from less effective emergency physicians?
- How do you communicate with patients, and in what ways (if any) do you think this differs from less effective emergency physicians?

**Examination:**

- How do you conduct patient examinations, and in what ways (if any) do you think this differs from less effective emergency physicians?

**Laboratory and Imaging Modalities:**

- How do you use laboratory tests and imaging studies, and in what ways (if any) do you think this differs from less effective emergency physicians?

**Medical Record Management:**

- How do you manage reviewing and documenting medical records, and in what ways (if any) do you think this differs from less effective emergency physicians?
